# Moderate to vigorous physical activity participation associated with better quality of life among breast and colorectal cancer survivors in Korea

**DOI:** 10.1186/s12885-020-06819-z

**Published:** 2020-05-01

**Authors:** Ji-Hye Park, Dong Hoon Lee, Seung Il Kim, Nam Kyu Kim, Justin Y. Jeon

**Affiliations:** 1 Department of Sport Industry Studies, Yonsei University, 50 Yonsei-ro, Seodaemun-Gu, Seoul, South Korea; 2 Department of Nutrition, Harvard T.H. Chan School of Public Health, Boston, MA USA; 3 Department of Surgery, Yonsei University College of Medicine, Yonsei University, 50 Yonsei-ro, Seodaemun-Gu, Seoul, South Korea; 4 Frontier research institute of Convergence Sports Science, Yonsei University, Seoul, South Korea; 5 Cancer Prevention Center, Yonsei Cancer Center, Yonsei University College of Medicine, Seoul, South Korea; 6 The Institute of Convergence Science, Yonsei University, Seoul, South Korea

**Keywords:** Physical activity, Quality of life, Breast cancer, Colorectal cancer, Survivor, Korea

## Abstract

**Background:**

To investigate the association between physical activity (PA) and quality of life (QoL) among breast and colorectal cancer survivors in Korea.

**Methods:**

A total of 224 cancer survivors (151 breast and 73 colorectal cancers) who completed treatments were recruited. We measured PA level with Godin Leisure-Time Exercise Questionnaire and QoL with European Organization for Research and Treatment of Cancer (EORTC) QLQ C-30.

**Results:**

Moderate to vigorous PA was significantly correlated with global QoL (*r* = .311, *p* < 0.01), physical functioning (*r* = .231, *p* < 0.01), fatigue (*r* = −.176, *p* < 0.05), pain (*r* = −.154, *p* < 0.05), and dyspnea (*r* = −.221, *p* < 0.01) while no correlation was found between light PA and QoL after controlling for potential confounders. When we further divided our participants into four groups by total PA level, we found a strong linear dose-response relationship between higher total PA and better QoL outcomes (*p* < .001). Compared with participants in the lowest quartile, those in the highest quartile had significantly better score in global QoL (65.8 ± 2.7 vs. 77.6 ± 2.8, *p* = 0.003), physical functioning (67.2 ± 2.3 vs. 85.3 ± 2.4, *p* = 0.007), fatigue (35.9 ± 3.2 vs. 23.6 ± 3.2, *p* = 0.008), pain (22.7 ± 3.3 vs. 13.0 ± 3.4, *p* = 0.046), and dyspnea (13.7 ± 2.5 vs. 5.9 ± 2.6, *p* = 0.034).

**Conclusions:**

Higher PA level was associated with better QoL among breast and colorectal cancer survivors in Korea. Increasing PA levels should be included as one of important strategies to improve QoL in cancer survivors.

## Background

Over 1 million cancer survivors are alive in Korea in 2016 [1] and cancer has been the leading cause of death in Korea since 1983 and is one of the most serious diseases [2]. Overall, the 5-year relative survival rate for people diagnosed with cancer between 2012 and 2016 was 70.6%, which represents an improved survival rate as compared with 41.2% for people diagnosed between 1993 and 1995 [1]. Cancer survivors report significantly lower levels of health-related quality of life (QoL) than non-cancer population [3, 4]. Cancer diagnosis is a major stressor resulting in considerable psychological suffering [5–7]. During and after cancer treatments, cancer patients frequently experience diverse physical and psychological symptoms including anxiety, fear, fatigue, pain, depression as well as decreases in overall functions [8–10]. Additionally, previous studies in diverse populations indicate that suicide is approximately twice as prevalent among patients with cancer compared with the general population [11–13].

Well-known way to improve mental health and QoL among cancer patients is through exercise and physical activity (PA). A systemic review reported that PA interventions significantly improved QoL in cancer survivors [14, 15]. Additionally, PA participation has been associated with reduced cancer-specific and all-cause mortality in breast and colorectal cancer survivors [16, 17]. Despite the growing evidence showing the benefits of exercise and PA on physical function, psychological health and prognosis and the safety of participating in exercise for cancer patients, many cancer patients still remain physically inactive [16, 18, 19]. Studies reported that 30–47% of cancer survivors in the United States met the American College of Sports Medicine (ACSM)‘s exercise recommendation [20, 21], while only 25.2% of Korean colorectal cancer survivors met the ACSM’s exercise recommendations that is significantly lower than the percentage among the non-cancer population [22]. Although the benefit of PA is well known among cancer survivors, there is limited data on the relationship between PA participation and QoL among Korean cancer survivors. Therefore, the purpose of this study was to investigate the association between the level of self-reported PA and QoL in breast and colorectal cancer survivors in Korea.

## Methods

### Study design

Potential participants were screened for eligibility via a medical record review before their arrival at the clinic. Upon arrival at the clinic, oncologists asked patients if they were willing to participate in a study. The research coordinator explained the study in detail and obtained a written consent. Each participant completed PA and QoL questionnaires. This research was a cross-sectional study conducted at Shinchon Severance Hospital Cancer Clinic, in Seoul, Korea. The study was approved by the Institutional Review Board of the Yonsei University College of Medicine.

### Participants and procedure of the study

The eligibility criteria for the study were: (1) aged over 18 years old, (2) completed primary and adjuvant treatments for colorectal and breast cancer (stage 0-IV), (3) ability to read and speak Korean. Participants who had any of the following characteristics or disorders were excluded from the study: participants who had a prior history of any cancer (except breast and colorectal cancer), current psychiatric illness, cardiovascular disease and/or diabetes, or had any other condition (e.g. neurological, orthopedic disorders) that made them unsuitable for participation in this study. Based on prior literature [23], we used G*Power to calculate required study sample size to detect small differences in mean QoL scores across physical activity groups given 80% power in a two-sided test with α-level of 0.05. In the current study, a total of 232 breast and colorectal cancer patients were initially recruited and screened for eligibility between 2013 and 2014. Among these participants, we excluded 8 cancer patients who had a prior history of other cancers and 224 eligible cancer patients (96.6%) agreed to participate in the study and completed PA and QoL questionnaires. The final analysis included 224 breast and colorectal cancer patients.

### Main outcome measurement

#### PA questionnaire

The amount of PA participation was assessed by the leisure score index (LSI) using the Godin Leisure-Time Exercise Questionnaire [24, 25]. Participants were asked to report their average weekly frequency and duration of light, moderate, or vigorous intensity exercise. The weekly exercise intensity was categorized as follows: Light (3 metabolic equivalent task (MET)). Moderate (5 MET), and Vigorous (9 MET). Of note, a MET indicates the ratio of the rate of energy expenditure during a specific activity to the rate of energy expenditure at rest. The summary totals for each intensity time were calculated, along with the total exercise time within a week.

Based on the PA information, we also calculated the percentage of participants meeting the ACSM PA Guidelines for Americans for cancer survivors (≥150 min/wk. of moderate-intensity or ≥ 75 min/wk. of vigorous-intensity aerobic exercise or an equivalent combination of moderate- and vigorous intensity aerobic exercise). The ACSM PA guideline has been widely used in many countries, including Asian countries, for prevention of cancer and other diseases and the PA guideline has shown similar benefits for non-Asian and Asian individuals [26].

#### QoL questionnaire

QoL was assessed with the European Organization for Research and Treatment of Cancer (EORTC) QLQ C-30 instrument, which has been widely used to assess QoL of cancer survivors [27, 28]. It is a 30-item measure of QoL consisting of five multi-item functional scales (physical, role, emotional, cognitive, social), three multi-item symptom scales (fatigue, pain, nausea and vomiting), six single-item symptom scales (dyspnea, insomnia, appetite loss, constipation, diarrhea, and financial difficulties), and one multi-item QoL scale. The scores of scales range from 0 to 100, with higher scores representing higher QoL.

#### Statistical analysis

All data were analyzed using SPSS 21.0 software (SPSS Inc., Chicago, IL, USA). Descriptive statistics were used to present the demographic and medical characteristics and physical activity level of the participants. Spearman correlation analyses were used to test for a potential relationship between PA and QoL. For the primary analysis, we categorized participants into quartiles based on their total PA level. ANCOVA was used to examine the differences in QoL outcomes across the quartiles of total PA level after adjusting for important demographic and cancer treatment-related factors including age, BMI, gender, marital status, income, education, types of cancer, and time since surgery. These potential confounders were chosen a priori based prior literature on physical activity and quality of life in cancer survivors [14, 15]. We further conducted subgroup analyses to explore whether the association between PA and QoL differs by potential effect modifiers including age, BMI, sex, marital status, income, education, types of cancer, time since surgery. We classified participants into two groups based on the ACSM PA guideline for the subgroup analyses. Interactions were tested by including interaction terms for PA and the potential effect modifiers in the model. A multiple linear regression analysis was performed to determine the association between QoL and PA and demographic/treatment factors. In this model, QoL was included as a dependent variable and independent variables we considered were age, BMI, gender, marital status, income, education, types of cancer, time since surgery, and total PA time. *P*-values less than 0.05 were considered statistically significant.

## Results

### Characteristics of participants

The participant’s demographic and medical characteristics are shown in Table 1. There were 224 participants (45 males and 179 females; 151 breast and 73 colorectal cancer patients) who completed questionnaires. Mean age was 54.4 ± 7.8 years old for males and 51.5 ± 8.2 years old for females. All participants were diagnosed with stage 0 to IV breast or colorectal cancer. (Table 1).

**Table 1:** Characteristics of breast and colorectal cancer survivors (*N* = 224)

Variables	Male (*N* = 45)		Female (*N* = 179)		Total (*N* = 224)	
	N	%	N	%	N	%
Age						
< 60 yrs	34	15.2	146	65.2	180	80.4
≥ 60 yrs	11	4.9	33	14.7	44	19.6
BMI						
< 23 kg/m^2^	11	5.4	105	51.7	116	57.1
≥ 23 kg/m^2^	24	11.8	63	31	87	42.9
Material status						
Married	44	19.7	148	66.4	912	86.1
Single	0	0	9	4.0	9	4.0
Widowed	0	0	8	3.6	8	3.6
Divorced	1	0.4	13	5.8	14	6.3
Average monthly household income status						
≤ $1000	1	0.5	21	9.6	22	10.1
$1001–$3000	15	6.9	53	24.3	68	31.2
$3001–$5000	14	6.4	61	28	75	34.4
≥ $5001	15	6.9	38	17.4	53	24.3
Education						
Middle school graduate or less	5	2.2	35	15.7	40	17.9
High school gradate	18	8.1	75	33.6	93	41.5
University/college	14	6.3	53	23.8	67	30
Higher degree than university/college	8	3.6	15	6.7	23	10.3
Occupation						
Professional/business	13	5.8	11	4.9	24	10.8
Office	3	1.3	10	4.5	13	5.8
Sale/Technical	3	1.3	8	3.6	11	4.9
Production/Labor	0	0	2	0.9	2	0.9
Service	4	1.8	11	4.9	15	6.7
Self employed	8	3.6	19	8.5	27	12.1
Education/government	3	1.3	2	0.9	5	2.2
Housewife	0	0	91	40.8	91	40.8
Unemployed	9	4.0	18	8.1	27	12.1
Other	2	0.9	6	2.7	8	3.6
Types of cancer						
Breast cancer	0	0	151	67.4	151	67.4
Colorectal cancer	45	20.1	28	12.5	73	32.6
Stages of caner						
Stage 0	0	0	3	1.4	3	1.4
Stage I	16	7.7	75	36.2	91	44.0
Stage II	9	4.3	58	28	67	32.4
Stage III	14	6.8	26	12.6	40	10.3
Stage IV	3	1.4	3	1.4	6	2.9
Time since surgery						
< 2 yrs	26	12.0	86	39.8	112	51.9
≥ 2 yrs	19	8.8	85	39.4	104	48.1

*Abbreviation: BMI* body mass index

The average weekly total PA participation was 346.1 ± 316.6 min while PA of male participants were substantially higher than female participants (449.5 ± 397.6 vs 320.1 ± 288.3). Sixty three out of 224 (27.7%) cancer survivors met the ACSM PA guidelines for cancer survivors (Table 2).

**Table 2:** Physical activity levels of breast and colorectal cancer survivors in Korea

Variables	Mean (SD)	Median (IQR)
**Male (*N*** **= 45)**		
PA time (min/week)		
Vigorous intensity PA	95.1 ± 201.5	0 (0–120)
Moderate intensity PA	206.6 ± 367.1	0 (0–280)
Light intensity PA	147.8 ± 189.5	30 (0–270)
Total PA	449.5 ± 397.6	385 (210–630)
Meeting the ACSM PA guideline, n (%)	27 (12.1)	–
**Female (N = 179)**		
PA time (min/week)		
Vigorous intensity PA	9.3 ± 45.1	0 (0–0)
Moderate intensity PA	80.3 ± 214.6	0 (0–0)
Light intensity PA	230.5 ± 109.7	180 (75–330)
Total PA	320.1 ± 288.3	270 (135–420)
Meeting the ACSM PA guideline, n (%)	35 (15.6)	–
**Total (N = 224)**		
PA time (min/week)		
Vigorous intensity PA	26.5 ± 104.0	0 (0–0)
Moderate intensity PA	105.7 ± 256.8	0 (0–61)
Light intensity PA	213.0 ± 208.1	180 (0–309)
Total PA	346.1 ± 316.6	290 (143–450)
Meeting the ACSM PA guideline, n (%)	63 (27.7)	–

*Abbreviation: ACSM* American College of Sport Medicine, *IQR* interquartile range, *PA* physical activity, *SD* standard deviation

ACSM PA guideline: ≥150 min/wk. of moderate-intensity or ≥ 75 min/wk. of vigorous-intensity aerobic exercise or an equivalent combination of moderate- and vigorous intensity aerobic exercise

### Relationship between PA participation and QoL

To examine the association between the amount of PA participation and QoL, we studied whether the amount of PA and overall QoL as well as functional and symptom scale of QoL would be associated according to the intensity of PA; moderate to vigorous PA, light PA and total PA with and without adjustment for potential confounders. Overall QoL was significantly associated with the amount of moderate to vigorous PA and total PA with and without adjustment of potential confounding variables.

The amount of moderate to vigorous PA participation was significantly correlated with physical functioning (*r* = 0.292, *p* < 0.01), emotional functioning (*r* = 0.195, *p* < 0.01), cognitive functioning (*r* = 0.175, *p* < 0.01), fatigue (*r* = −.263, *p* < 0.01), pain (*r* = −.220, *p* < 0.01), dyspnea (*r* = −.176, *p* < 0.01), insomnia (*r* = −.132, *p* < 0.05), appetite loss (*r* = −.188, *p* < 0.01), and constipation (*r* = −.144, *p* < 0.05). When correlation analyses were further performed after adjustment for potential confounding factors, the amount of moderate to vigorous PA participation was still significantly correlated with physical functioning (*r* = .231, *p* < 0.01), emotional functioning (*r* = 0.155, *p* < 0.05), fatigue (*r* = −.176, *p* < 0.05), pain (*r* = −.154, *p* < 0.05), and dyspnea (*r* = −.221 *p* < 0.01) (Table 3).

**Table 3:** Spearman correlation between physical activity levels and quality of life (EORTC QLQ C-30) in breast and colorectal cancer survivors in Korea

		Intensity of PA			Intensity of PA (adjusted)^a^		
		Moderate to vigorous PA	Mild PA	Total PA	Moderate to vigorous PA	Mild PA	Total PA
QoL		.267**	−.007	.231**	.311**	−.031	.249**
Functional scales	Physical functioning	.292**	.029	.277**	.231**	.082	.251**
	Role functioning	.117	.096	.166*	.043	.159*	.136
	Emotional functioning	.195**	−.050	.139*	.155*	−.030	.115
	Cognitive functioning	.175**	−.049	.122	.106	−.040	.067
	Social functioning	.023	−.010	.014	−.030	−.023	−.041
Symptom scales	Fatigue	−.263**	−.064	−0.275**	−.176*	−.094	−.210**
	Nausea and vomiting	−.071	−.011	−.070	.002	−.020	−.011
	Pain	−.220**	.060	−.155*	−.154*	−.022	−.147
	Dyspnea	−.176**	.075	−.106	−.221**	−.024	−.206**
	Insomnia	−.132*	−.032	−.138*	−.117	−.076	−.149
	Appetite loss	−.188**	.042	−.138*	−.126	−.016	−.119
	Constipation	−.144*	−.029	−.146*	−.139	.013	−.113
	Diarrhea	.112	−.149*	.002	.114	−.131	.042
	Financial difficulties	−.041	−.022	−.051	−.072	−.003	−.064

*Abbreviation: EORTC QLQ*, European Organization for Research and Treatment of Cancer Quality of Life Questionnaire, *PA* physical activity, *QoL* quality of life

^a^Adjusted for age, BMI, sex, marital status, income, education, cancer type, stage of cancer, time since surgery, *P < .05, **P < 0.01

To further analyze the relationship between the amount total PA participation and QoL, participants were divided into four groups (Quartile) and their QoL were compared. Overall, we found a strong linear dose response between higher PA and better QoL in cancer survivors (*p* < .001). Compared with participants in the lowest quartile of total PA, participants in the higher quartile of PA had significantly higher scores in global QoL (Quartile 1: 65.8 ± 2.7 vs. Quartile 4: 77.6 ± 2.8*, p* = 0.003) and physical functioning (Quartile 1: 67.2 ± 2.3 vs. Quartile 4: 85.3 ± 2.4*, p* = 0.007) while significantly lower scores in fatigue (Quartile 1: 35.9 ± 3.2 vs. Quartile 4: 23.6 ± 3.2*, p* = 0.008), pain (Quartile 1: 22.7 ± 3.3 vs. Quartile 4: 13.0 ± 3.4*, p* = 0.046) and dyspnea (Quartile 1: 13.7 ± 2.5 vs. Quartile 4: 5.9 ± 2.6*, p* = 0.034) after adjustment of potential confounding variables (Supplementary Table 1).

For the subgroup analyses, we divided our participants into two groups; who meet the ACSM PA guideline and do not meet the ACSM PA guideline. In these analyses, we further divided our participants by potential effect modifiers and compared their QoL between subgroups. Compared with participants diagnosed with stage 0-I cancer, meeting the ACSM PA guideline was more strongly associated with better QoL among participants diagnosed with stage II-IV cancer (P for interaction = 0.04). Overall, participants who met the ACSM PA guideline had better QoL, regardless of age, gender, BMI, type of cancer, time since surgery, monthly household income and education level (Table 4).

**Table 4:** Adjusted mean (95% CI) of quality of life (QoL) according to the ACSM’s physical activity guideline by subgroups of breast and colorectal cancer survivors in Korea

	ACSM PA guideline		*P* interaction
	Not meeting	Meeting	
Age			
< 60 yrs	65.4 (61.9–69.0)	79.2 (73.4–84.9)*	0.42
≥ 60 yrs	72.0 (64.8–79.1)	78.3 (64.6–92.1)	
Gender			
Male	64.5 (53.1–76.0)	70.8 (60.7–80.8)	0.15
Female	67.8 (64.3–71.2)	82.2 (75.1–89.3)*	
BMI			
< 23 kg/m^2^	66.0 (61.9–67.0)	82.7 (63.4–73.3)*	0.12
≥ 23 kg/m^2^	68.3 (63.4–73.3)	75.7 (68.3–83.1)	
Types of Cancer			
Breast Cancer	66.3 (62.1–70.5)	80.1 (72.6–87.5)*	0.33
Colorectal Cancer	68.6 (61.7–75.5)	77.4 (65.2–89.5)	
Stages of cancer			
≤ Stage II	67.7 (64.1–71.4)	76.6 (70.9–82.4)*	0.04
> Stage II	63.8 (57.7–70.0)	90.0 (78.0–101.9)*	
Time since surgery			
< 2 yrs	66.7 (62.4–70.9)	76.1 (68.7–83.5)*	0.27
≥ 2 yrs	67.1 (62.5–71.7)	82.2 (74.5–90.0)*	
Average monthly household income status			
≤ $3000	63.1 (58.0–68.2)	77.6 (65.3–73.9)*	0.54
> 3000	69.6 (65.3–73.9)	80.0 (73.1–86.9)*	
Education			
≤ High school graduate	66.1 (61.9–70.2)	80.9 (74.0–87.7)*	0.21
> High school graduate	68.2 (63.8–73.5)	76.1 (67.8–84.4)	

*Abbreviation: ACSM* American College of Sport Medicine, *BMI* body mass index, *PA* physical activity

All models were adjusted for age, BMI, gender, marital status, income, education, types of cancer, time since surgery, *Significantly different from Not meeting ACSM guideline (*P* < 0.05)

Multiple regression analyses with QoL as dependent variable and gender, age, types of cancer, stage, time since surgery, income, education, BMI and total PA as independent variables showed that only total PA amount was a significant predictor of QoL ( *=* 0.25*, p =* < 0.01) (Table 5).

**Table 5:** Multiple regression analysis of quality of life as a dependent variable

Dependent variable	Variables		SE	Standardized	*P*-value
QoL	Gender	2.58	5.16	.06	.44
	Age	−.14	.18	−.06	.43.
	Types of cancer	1.62	4.45	.07	.39
	Stages of cancer	−2.16	1.59	−.11	.18
	Time since surgery	.04	.09	.04	.61
	Income	1.52	.84	.16	.07
	Education	.19	1.92	.01	.92
	BMI	.38	.45	.07	.45
	Total PA	.01	.00	.25	< 0.01

*Abbreviation: BMI* body mass index, *PA* physical activity, *QoL* quality of life, *SE* standard error

Gender, age, BMI, marital status, income, education, types of cancer, time since surgery, total PA count for quality of life by 8.6% (R^2^ = 0.043, F-value = 0.05)

## Discussion

This cross-sectional study was to examine whether the amount of self-reported PA is associated with QoL among Korean breast and colorectal cancer survivors. As hypothesized, higher amount of moderate to vigorous and total PA (sum of light, moderate, and vigorous PA) were significantly associated with higher QoL while no association between the amount of light PA and any of the QoL variables was shown. These associations were supported when our participants were divided into quartiles and their QoL outcomes were compared across quartiles. Compared with participants in the 1st quartile (the least active), participants in the 4th quartile had 11.8 point (65.8 vs. 77.6 point) higher scores in global QoL, clinically meaningful moderate differences [29] .

Findings from the current study are supported by previous studies reported that moderate to vigorous PA is associated with health-related QoL among colorectal cancer survivors [30, 31]. Moreover, accumulating evidence suggests that participation of PA contributes to improved QoL through positive changes in physical, psychological, social and spiritual factors [32, 33]. What is unique and interesting about our finding is that light PA was not associated with any of QoL variables. We have previously demonstrated that under cancer treatment, moderate to vigorous PA participation decreases while light PA increases among Korean colorectal cancer survivors [22]. Current ACSM PA recommendations for cancer survivors focus on accumulating adequate level of moderate to vigorous PA level (overall level of weekly activity of 150 min of moderate-intensity exercise or 75 min of vigorous-intensity exercise or an equivalent combination). Cochrane Review revealed that PA at moderate to vigorous intensity provides greater health benefits than low-intensity PA [34]. Interestingly, our study found that significantly higher QoL score in the 4th quartile of PA group compared with 1st quartile of PA group. Participants in the 4th quartile may have been only group which met the ACSM guidelines for cancer survivors (mean of moderate to vigorous PA Q1: 5.5 min/wk., Q2: 31.4 min/wk., Q3: 109.1 min/wk., Q4: 368.9 min/wk) but we still observed better QoL with higher PA in the second and third quartiles. Therefore, it is important to encourage moderate to vigorous PA among cancer survivors to improve QoL.

In order to better understand our data, we conducted subgroup analyses by potential effect modifiers and found that meeting the ACSM PA guideline was more closely associated with better global QoL among participants whose cancer stage was greater than stage II. Normally, cancer patients with stage 0-II undergo surgery and some receive additional therapy but cancer patients above stage II most often receive radiation and/or chemotherapy in addition to surgery. Patients undergoing radiation and chemotherapy treatment experience decline in their perceived QoL during treatment. This finding suggests that cancer survivors above stage II could benefit more from participating PA to improve QoL.

Given the importance of regular PA for disease prevention and health promotion [35], the low number of participants meeting the ACSM PA guideline is of concern. Our study found only 27.7% of cancer survivors met the exercise recommendations of the ACSM. Consistent with our findings, Chung et al. reported that 25.2% of Korean colorectal cancer patients met the ACSM PA guideline [22]. However, other studies have reported higher percentage of cancer survivors who met the ACSM PA guideline in other populations. Irwin et al., [18] reported 32% of breast cancer survivors and Blanchard et al. reported, [36] 30–47% of cancer survivors met the ACSM PA guideline. Recently, we have reported that oncologists’ PA recommendation to their cancer survivors when given with pedometer and exercise diary significantly increased cancer survivors’ PA participation [37]. Therefore, oncologists should team up with exercise specialists to provide proper and effective strategies to increase PA level of cancer survivors.

One of the limitations of our study is the nature of cross-sectional study that we cannot draw cause and effect conclusion. We may not be able to say higher PA level resulted in improved QoL since we cannot eliminate the possibility that those with higher QoL might have been in better physical condition which make them possible to participate in moderate to vigorous PA. To reduce this concern, we further analyzed our data after controlling for important sociodemographic and treatment related confounders and still found significant associations between the amount of PA and QoL. Furthermore, our multiple regression analysis also showed that only PA was a significant predictor of global QoL, suggesting the importance of PA. However, we suggest the need to further investigate the effect of moderate to vigorous exercise on QoL in a large randomized controlled trial among Korean cancer survivors. There are other limitations in our study. The reliance on self-report rather than objective measure of exercise behaviors may lead to imprecise measurements. However, use of accelerometer may also have limitation that predefined moderate to vigorous PA measured by accelerometer may not truly reflect PA levels of cancer survivors [38]. Moreover, our study sample was single clinic-based rather than population-based. Our study participants (particularly colorectal cancer patients) were younger than the general Korean cancer patients [39] which may reduce the generalizability of the findings to all breast and colorectal cancer survivors.

In conclusion, we found that increased moderate to vigorous PA participation was associated with higher QoL in breast and colorectal cancer survivors in Korea while no association was found between light PA and QoL. When cancer survivors can safely participate in PA with higher intensity, moderate PA should be recommended to improve QoL.

## Supplementary information

**Additional file 1: Table S1.** Adjusted mean of quality of life (QoL) factors across quartiles of total physical activity levels in breast and colorectal cancer survivors in Korea.

## Data Availability

The datasets used and/or analysed during the current study are available from the corresponding author on reasonable request.

## Abbreviations

ACSM: American College of Sports Medicine
BMI: Body mass index
EORTC: European Organization for Research and Treatment of Cancer
PA: Physical activity
QoL: Quality of life
