# Supplementary material for: Moderate to vigorous physical activity participation associated with better quality of life among breast and colorectal cancer survivors in Korea
Source: BMC Cancer. 2020 May 1;20:365. doi: 10.1186/s12885-020-06819-z (PMC7193341; doi:10.1186/s12885-020-06819-z)
Supplement: Supplementary file 1 — Additional file 1: Table S1. Adjusted mean of quality of life (QoL) factors across quartiles of total physical activity levels in breast and colorectal cancer survivors in Korea. [file 12885_2020_6819_MOESM1_ESM.docx]

**Supplementary Materials**

| **Supplementary Table 1. Adjusted mean of quality of life (QoL) factors across quartiles of total physical activity levels in breast and colorectal cancer survivors in Korea** | | | | | |
| --- | --- | --- | --- | --- | --- |
| Mean±SD | | Quartile 1  N=56  (M=10, F=46) | Quartile 2  N=56  (M=7, F=49) | Quartile 3  N=53  (M=12, F=41) | Quartile 4  N=59  (M=16, F=43) |
| QoL | | 65.8±2.7 | 67.9±2.8 | 68.9±2.8 | 77.6±2.8*#+ |
| Functional scales | Physical functioning | 67.2±2.3 | 78.0±2.35 | 79.0±2.41 | 85.3±2.35*# |
|  | Role functioning | 77.2±3.4 | 82.4±3.50 | 89.9±3.58* | 86.9±3.49 |
|  | Emotional functioning | 75.7±2.9 | 82.1±2.96 | 81.1±3.03 | 80.8±2.96 |
|  | Cognitive functioning | 81.1±2.7 | 81.2±2.75 | 81.3±2.82 | 78.7±2.75 |
|  | Social functioning | 88.4±3.5 | 81.9±3.62 | 86.8±3.70 | 84.2±3.61 |
| Symptom scales | Fatigue | 35.9±3.2 | 30.3±3.2 | 32.0±3.3 | 23.6±3.2* |
|  | Nausea and vomiting | 2.9±1.6 | 5.0±1.6 | 5.4±1.7 | 5.2±1.6 |
|  | Pain | 22.7±3.3 | 18.8±3.4 | 18.8±3.4 | 13.0±3.4* |
|  | Dyspnea | 13.7±2.5 | 10.7±2.6 | 12.4±2.3 | 5.9±2.6* |
|  | Insomnia | 31.1±4.8 | 23.4±4.9 | 32.2±5.0 | 19.4±4.9 |
|  | Appetite loss | 13.8±3.0 | 14.1±3.1 | 12.5±3.2 | 9.1±3.1 |
|  | Constipation | 19.8±4.3 | 20.5±4.4 | 13.3±4.5 | 13.1±4.4 |
|  | Diarrhea | 7.3±2.1 | 11.0±2.2 | 2.5±2.3# | 7.9±2.2 |
|  | Financial difficulties | 11.9±3.7 | 20.1±3.8 | 12.1±3.8 | 13.9±3.7 |
| Physical activity levels (min/wk): Quartile 1 ≤135, 135< Quartile 2 ≤280, 280< Quartile 3 ≤444, 444< Quartile 4.  Adjusted age, BMI, gender, marital status, income, education, types of cancer, time since surgery, *Significantly different from group1, # significantly different from group2, + significantly different from group3 (p<0.05) | | | | | |
